# Relating microbial community structure to functioning in forest soil organic carbon transformation and turnover

**DOI:** 10.1002/ece3.969

**Published:** 2014-02-12

**Authors:** Yeming You, Juan Wang, Xueman Huang, Zuoxin Tang, Shirong Liu, Osbert J Sun

**Affiliations:** 1 Ministry of Education Key Laboratory for Silviculture and Conservation, College of Forest Science, Beijing Forestry UniversityBeijing, 100083, China; 2 Institute of Forestry and Climate Change Research, Beijing Forestry UniversityBeijing, 100083, China; 3 State Forestry Administration of China Key Laboratory of Forest Ecology and Environment, Institute of Forest Ecology, Environment and Protection, Chinese Academy of ForestryBeijing, 100091, China

**Keywords:** Decomposition, extracellular enzymes, forest soil carbon, pathway analysis, phospholipid fatty acids (PLFAs), redundancy analysis (RDA), temperate forest

## Abstract

Forest soils store vast amounts of terrestrial carbon, but we are still limited in mechanistic understanding on how soil organic carbon (SOC) stabilization or turnover is controlled by biotic and abiotic factors in forest ecosystems. We used phospholipid fatty acids (PLFAs) as biomarker to study soil microbial community structure and measured activities of five extracellular enzymes involved in the degradation of cellulose (i.e., β-1,4-glucosidase and cellobiohydrolase), chitin (i.e., β-1,4-N-acetylglucosaminidase), and lignin (i.e., phenol oxidase and peroxidase) as indicators of soil microbial functioning in carbon transformation or turnover across varying biotic and abiotic conditions in a typical temperate forest ecosystem in central China. Redundancy analysis (RDA) was performed to determine the interrelationship between individual PFLAs and biotic and abiotic site factors as well as the linkage between soil microbial structure and function. Path analysis was further conducted to examine the controls of site factors on soil microbial community structure and the regulatory pathway of changes in SOC relating to microbial community structure and function. We found that soil microbial community structure is strongly influenced by water, temperature, SOC, fine root mass, clay content, and C/N ratio in soils and that the relative abundance of Gram-negative bacteria, saprophytic fungi, and actinomycetes explained most of the variations in the specific activities of soil enzymes involved in SOC transformation or turnover. The abundance of soil bacterial communities is strongly linked with the extracellular enzymes involved in carbon transformation, whereas the abundance of saprophytic fungi is associated with activities of extracellular enzymes driving carbon oxidation. Findings in this study demonstrate the complex interactions and linkage among plant traits, microenvironment, and soil physiochemical properties in affecting SOC via microbial regulations.

## Introduction

The interactions between above-and below-ground components play an important role in driving ecosystem processes, but their underlying mechanisms are yet poorly understood. Thus, there have been continued and growing interests to elucidate the explicit relationships between vegetation and below-ground processes and to seek mechanistic understanding on the contribution of soil biota and associated processes to ecosystem functioning (Wardle et al. 2004; Xiao et al. 2007; De Deyn et al. 2008; Jin et al. 2010; Zhou et al. 2013).

Soil microorganisms are a critical link between shifts in the composition of dominant vegetation and fundamental shifts in ecosystem functioning (Waldrop et al. 2000; Prescott and Grayston 2013; Prescott and Vesterdal 2013). Soil active microbial communities play a central role in below-ground processes, especially in mediating soil organic matter decomposition and nutrient cycling in forest ecosystems (van der Heijden et al. 2008). An improved knowledge on the interactions between site factors and soil microbial communities in facilitating forest soil organic matter decomposition and soil carbon sequestration is increasingly recognized as key to understanding feedbacks of terrestrial ecosystem processes to global climate change (Singh et al. 2010). However, owning to the complexity of below-ground processes as well as technical difficulties to experimentally manipulate soil microbial structures and activities, significant gaps remain in our current understanding on how soil microbial communities are controlled by complex interactions of biotic and abiotic site factors and how the structural shifts in soil microbial communities are linked to alterations of their functioning, such as in mediating soil organic carbon (SOC) dynamics (Hackl et al. 2005; Brockett et al. 2012). While advancement in biotechnology has allowed for the development of techniques for structural analysis of soil microbial communities, *for example,* the characterization of microbial functional components with phospholipid fatty acids (PLFA) analysis, determinations of the linkage between microbial structure and function still pose a critical issue (Prescott 2010). A lack of effective technical solution in directly relating microbial community structure to functioning in SOC transformation and turnover in natural ecosystems has been an obstacle for seeking mechanistic understanding on the feedbacks of changes in below-ground processes to ecosystem succession and functioning in response to climate and land-cover changes.

The PLFAs extracted from soil are used as an indicator of microbial community structure, as certain groups of microorganisms have different “signature” fatty acids (Tunlid and White 1992). These lipids are only present in the membranes of viable organisms and are rapidly degraded in soil and as such are a measure of the organisms living at the time of sampling (Grayston and Prescott 2005). A more advanced technique based on nucleic acid extraction and analysis has also been increasingly used for examining soil microbial community structure, particularly genes coding for ribosomal RNA (rRNA). However, the PLFA method is a rapid and inexpensive way of assaying the biomass and composition of microbial communities in soils and may even be more sensitive in detecting shifts in microbial community composition when compared to nucleic acid-based methods (Ramsey et al. 2006). Although the PLFA method cannot compete with the rRNA methods in the phylogenetic resolution by which a given community can be characterized, the latter provide little information on the phenotype and the activity of the microorganisms in the environment (Frostegård et al. 2011).

Soil microbial community structure in natural ecosystems is known to be directly influenced by many edaphic factors, such as temperature, water, and soil physicochemical properties (Williams and Rice 2007; Collins et al. 2008; Angel et al. 2010; Castro et al. 2010). Vegetation type and structure, by modifying the site microclimate, the quantity and quality of litter, the production of root exudates, and/or the allocation patterns of organic matter, directly and indirectly affect soil microbial community structure (De Deyn et al. 2008; Wardle et al. 2012; Jassey et al. 2013). Previous studies have demonstrated that ecosystems may differ in the relative influences of site factors on soil microbial communities. For example, Brockett et al. (2012) found that soil water was the dominant factor influencing the potential function of the soil microbial community across forests of varying biogeoclimatic zones. In contrast, many studies show that SOC is closely related to soil microbial community structure and function under different types of vegetation (Grayston and Prescott 2005; Yao et al. 2006; Franklin and Mills 2009; Katsalirou et al. 2010). There are also findings that specific soil chemical properties, such as soil C/N ratio (Fierer et al. 2009), nutrient status (Lauber et al. 2008), and soil pH (Rousk et al. 2009), are highly correlated with soil microbial community composition and function, and that plant litter chemistry (Ushio et al. 2008; Strickland and Rousk 2010) and spatial pattern of soil properties (Ushio et al. 2010) can impose marked impacts on forest soil microbial function by changing soil microbial community composition. Despite a much improved understanding on the controls of below-ground processes, however, there lacks a general model quantifying the relative contributions to, and the levels of control of, soil microbial processes when multiple biotic and abiotic site factors are involved. The underlying mechanisms on the functional linkages between plant communities and soil microbial communities and the impacts on forest biogeochemical processes that arise as a consequence of below-ground activities are much complicated by the multiple effects of various site factors, biotic or abiotic, leaving alone the complexity in interactions and autocorrelations among the factors that potentially lead to nonlinearity of biotic and environmental controls of ecosystem processes. Clearly, a holistic approach taking into consideration of all potential factors and drivers is necessary when examining the structure–function relationships of soil microbial communities in order to gain mechanistic understandings on the controls and functioning of below-ground processes.

**Figure 1:**
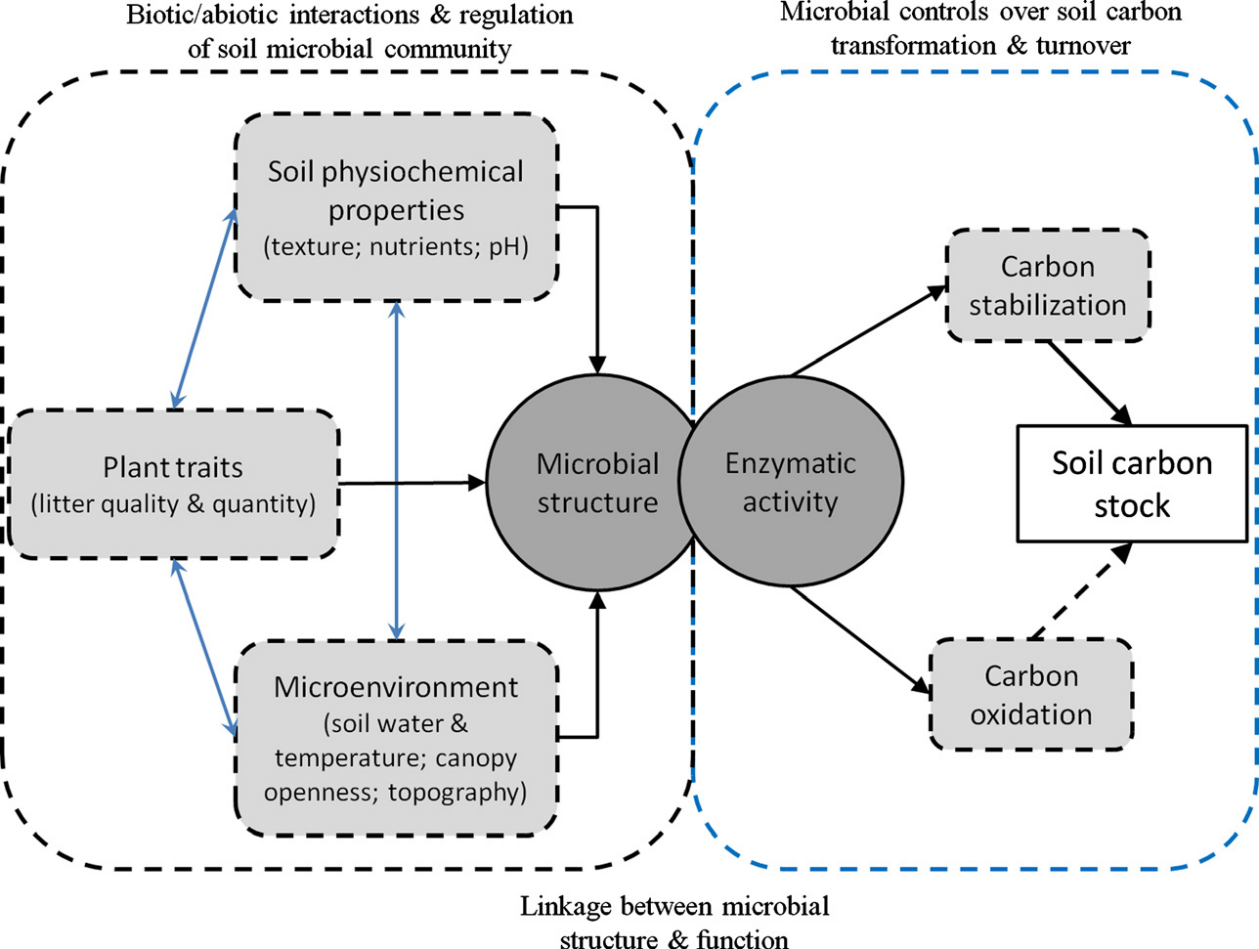
A conceptual model illustrating the interactions between site biotic and abiotic factors and regulations of soil microbial community, linkage between soil microbial structure and function, and microbial controls over soil carbon stabilization and turnover via effects on enzymatic activities.

In this study, using a typical temperate forest in central China as a model ecosystem, we examined the linkage between soil microbial structure and functioning in driving SOC transformation and turnover and assessed the relative influences of biotic and abiotic site factors on soil microbial structure and function. Soil microbial community structure, as characterized by PLFA analysis, and microbial function in driving SOC transformation and turnover, as represented by five extracellular enzymes involved in the degradation of cellulose (i.e., β-1,4-glucosidase and cellobiohydrolase), chitin (i.e., β-1,4-N-acetylglucosaminidase), and lignin (i.e., phenol oxidase and peroxidase), were investigated across varying biotic and abiotic conditions established by setting up sampling plots in different types of forest stands naturally occurring or historically planted in the study area. Multivariate analysis and pathway analysis were used to identify the dominant biotic and abiotic site factors affecting soil microbial structure and function and to determine the regulatory pathway of changes in SOC relating to microbial community structure and function. Our objectives were to determine that, in forest ecosystems, (1) how soil microbial communities are controlled by complex interactions of biotic and abiotic site factors; and (2) how and to what extend the structural variations in soil microbial communities are linked to functioning in driving SOC transformation and/or turnover. We anticipate that the outcome of the study would help with gaining better mechanistic understanding on the microbial regulation of SOC dynamics. We hypothesized that soil microbial community controls SOC stabilization or turnover through a linkage between structure and function and that the role of biotic and abiotic site factors in affecting SOC stock is operated via regulations on the structural attributes of soil microbial community (Fig. 1).

## Materials and Methods

### Study sites

The study was located in field sites at the Baotianman Long-Term Forest Ecosystem Research Station in the Baotianman Nature Reserve (latitude 33°20′–33°36′N, longitude 111°46′–112°04′E, and elevation 600–1860 m a.s.l.), in the east of the Qinling Mountains in central China. It is in a transitional zone from warm temperate to northern subtropical climatic region. The climate is of a continental eastern monsoon type, with annual mean air temperature of 15.1°C and mean annual precipitation of 900 mm (Luan et al. 2011). Precipitation occurs mainly in the summer months June through August (55–62%; Liu et al. 1998). The soils are of dystric cambisols (FAO-UNESCO soil classification system) developed on weathered arenites.

The zonal vegetation at the study sites is typically a deciduous broadleaved forests dominated by, with changes in topography and position on slopes, *Quercus aliena* var. *acuteserrata* Maxim., *Quercus glandulifera* var. *brevipetiolata* Nakai., and *Quercus variabilis* Blume., respectively, in the canopy layer. *Pinus armandii* Franch. plantations were established in the region for timber production around 1956, but some stands developed into mixed forests due to natural regeneration of *Q. aliena* and implementation of forest protection schemes. There are also some azonal tree species, such as *Acer mono* Maxim., *Toxicodendron verniciflnum* (Stokes) F. Barkley, *Carpinus cordata* Bl., and *Populus davidiana* Dode, etc., occurring in low frequency in the local forests. The understory is composed of predominantly woody plants *Lauraceae obtusiloba* Bl., *Rubus corchorifolius* Linn. F, *Vitis amurensis* Rupr., *Carpinus turczaninowii* Hance., *Celastrus orbiculatus* Thunb., and *Clematis florida* Thunb. in the shrub layer, with infrequent occurrence of *Pteridium aquilinum* (Linn.) Kuhn var. *latiusculum* (Desv.) Underw. ex Heller and highly sparse herbaceous plants *Carex siderosticta* Hance., *Carex lanceolata* Boott, *Rodgersia aesculifolia* Batal., *Duchesnea indica* (Andr.) Focke, and *Adenophora axilliflora* Borb. in the field layer.

Four forest types, each dominated by *Q. aliena*,*Q. glandulifera*,*Q. variabilis*, and mixture of *P. armandii* and *Q. aliena*, respectively, and an age sequence of *Q. aliena* forest (∼40, ∼80, and >160 years), were included in this study. Three 20 m × 20 m plots were set up on separate sites for each of the forest types and the age sequence stands in April 2011. The stands of similar age (∼80 years) were selected for forest types representing *Q. aliena*,*Q. glandulifera*, and *Q. variabilis*. The mixed *Q. aliena* and *P. armandii* forest stands were aged >50 years. Stand age was obtained from forest management records and increment core samples. All sites are under natural conditions and have not experienced apparent anthropogenic disturbance in recent history.

Field survey was conducted on each plot to collect data on tree species composition and density, size of individual trees > 5 cm DBH, canopy openness, litter mass on forest floor, fine root biomass, and soil physicochemical properties.

### Field measurements and sampling

Soil temperatures at 10 cm depth (*T*_soil10_) were measured using HOBO Data Loggers (TidbiT v2 Temp, Onset Computer Corporation, Cape Cod, MA) at 30 min intervals. The average soil temperature for each plot was calculated for 4 weeks prior to soil sampling, which represented the critical time period for microbial response to varying environmental conditions (Bell et al. 2009). Plant fine roots and litter samples were collected following soil sampling. Litter samples (including undecomposed plant tissues, partially decomposed duff, and *O*_e_ and *O*_a_ horizons) were collected on each plot at 12 random locations with a 20 cm diameter ring frame and oven-dried at 65°C to constant weight. Twelve root core samples were also collected on each plot by soil layer for 0–5 cm, 5–10 cm, and 10–20 cm using a 15-cm-inner-diameter soil-corer. They were washed clean using 0.4-mm mesh bags and separated into coarse (>5 mm), medium (2–5 mm), and fine (<2 mm) roots before being oven-dried at 65°C to constant weight. All litter and root samples were measured for total dry mass and analyzed for concentrations of C and N, and the acid soluble fraction and insoluble residue (i.e., the “Klason lignin”) (Parton et al. 2007; Meier and Bowman 2010).

Soil samples were collected in mid-August 2012, at 12 systematically arranged locations on each plot at a distance of 5 m from the plot center in the directions of 0, 30, 60, 90, 120, 150, 180, 210, 240, 270, 300, and 330°. They were collected at depths of 0–5 cm, 5–10 cm, and 10–20 cm using a 10-cm-inner-diameter soil-corer. The samples were mixed to obtain one composite sample for each of the soil layers on each plot. All soil samples were immediately stored on ice in insulated containers upon collection and sieved to pass a 2-mm mesh after returning to laboratory. Each processed soil sample was divided into two portions and stored in a refrigerator at −20°C before being analyzed. One was freeze-dried for PLFA analysis and the other for enzyme assays and physicochemical analysis.

Altogether, 216 forest floor litter samples, 648 fine root samples, and 54 composite soil samples were collected and analyzed in this study.

### Measurements of soil physicochemical properties

The gravimetric soil water content (% SWC) was calculated from the mass loss after drying the samples at 105°C to a constant weight, for at least 48 h. We used a hydrometer method for analysis of soil particle size distribution (Sheldrick and Wang 1993). The soil pH was measured by mixing the soil sample with deionized water at a 1:2.5 ratio (w/v; Liu et al. 2012). The supernatants were measured using a pH meter (HI-9125, Hanna Instruments Inc, Woonsocket, RI). Soil organic carbon (SOC) content was analyzed by K_2_Cr_2_O_7_-H_2_SO_4_ calefaction method (Nelson and Sommers 1982). Soil total nitrogen (TN) content was analyzed using Kjeldahl digestion procedure (Gallaher et al. 1976). Soil ammonium nitrogen (NH_4_-N) and nitrate nitrogen (NO_3_-N) were determined from 10 g (dry mass) of soil using 50 mL 2M KCl extraction procedure and analyzed colorimetrically by a continuous flow analyzer (SEAL AA3, Norderstedt, Germany). Microbial biomass carbon (MBC) was measured by fumigation–extraction method, using 0.5 mol/L K_2_SO_4_ as extracting agent; a conversion factors of 2.64 were used to convert extracted C to MBC (Vance et al. 1987). The dissolved organic carbon (DOC) was determined by directly measuring carbon concentration in the 0.5 mol/L K_2_SO_4_ extract solution without fumigation treatment (Wu et al. 2011).

### Phospholipid fatty acids (PLFA) analysis

Microbial community structure was assessed by analyzing the composition of extractable ester-linked PLFAs, using the method was described by Bossio and Scow (1998). Concentrations of individual PLFAs were calculated based on 19:0 internal standard concentrations. The indicator PLFAs were used for classification of microbial community types. Bacterial community was represented by PLFAs i14:0, 15:0, i15:0, a15:0, i16:0, 16:1ω7c, 17:0, a17:0, i17:0, cy17:0, 18:1ω7c, and cy19:0 (Frostegård and Bååth 1996; Liu et al. 2012). Gram-positive bacteria are composed of PLFAs i14:0, i15:0, a15:0, i16:0, a17:0, and i17:0 (Liu et al. 2012; Nie et al. 2013), Gram-negative bacteria 16:1ω7c, cy17:0, 18:1ω7c, and cy19:0 (Zelles 1999; Liu et al. 2012), actinomycete 10Me16:0, 10Me17:0, 10Me 18:0 (Frostegård and Bååth 1996; Bell et al. 2009; Brockett et al. 2012), saprotrophic fungi 18:2ω69c (Nie et al. 2013), and arbuscular mycorrhizal fungi (AMF) 16:1ω5c (Olsson 1999; Brockett et al. 2012; Nie et al. 2013). Other PLFAs such as 14:0, 16:0, 16:1 2OH, 16:1ω9c, 17:1ω8c, and 18:1ω9c were also used for analysis of the microbial composition (Liu et al. 2012).

### Soil extracellular enzyme assays

We assessed microbial function by measuring activities of five soil extracellular enzymes involved in degrading cellulose (β-1,4-glucosidase and cellobiohydrolase), chitin (β-1,4-N-acetylglucosaminidase), and lignin (phenol oxidase and peroxidase), respectively. The activities of β-glucosidase (BG; EC:3.2.1.21), cellobiohydrolase (CBH; EC:3.2.1.91), and N-acetyl-β-glucosaminidase (NAG; EC:3.2.1.30) were determined by the conventional *p*-nitrophenol assays (Parham and Deng 2000; Baldrian 2009).

Phenol oxidase and peroxidase activities were determined using 1-3,4-dihydroxyphenylalanine (L-DOPA) as substrate (Sinsabaugh et al. 1993; Li et al. 2010). For phenol oxidase, the reaction mixture composed of 2 mL 5 mmol/L L-DOPA solution and soil slurry (1 g fresh soil with 1.5 mL 50 mmol/L sodium acetate buffer), and peroxidase activity assays received 2 mL of 5 mmol/L-DOPA and soil slurry (1 g fresh soil with 1.5 mL 50 mmol/L sodium acetate buffer), plus 0.2 mL of 0.3% H_2_O_2_. All total enzyme activities were expressed as μmol/g soil/h. The results of all enzymatic assays were expressed on a dry-weight basis. There were three replicates for each soil sample.

The specific enzyme activity, which is defined as the activity of a given enzyme on per unit of SOC basis, was calculated for each of the five soil extracellular enzymes for comparison of their relative contributions to the dynamics of SOC decomposition (Baldrian et al. 2013).

### Soil microbial respiration measurements

Soil microbial respiration (MR) was measured by determining CO_2_ evolution over a 12-day incubation following the procedure described by Jin et al. (2010). Specifically, 20 g (dry weight equivalent) of each soil sample was adjusted to 60% of water-holding capacity (WHC) and placed in a 250-mL Mason jar. Respired CO_2_ was absorbed in 10 mL 0.1 mol/L NaOH solution suspended inside the jar. All jars were sealed with rubber septum and incubated at 25°C and for 12 days in the dark. The NaOH solutions containing dissolved CO_2_ were then each titrated with 0.05 mol/L HCl solution to determine the amount of CO_2_ evolved at 3-day intervals. After each reading, the jars were left open for half an hour to ventilate and then resealed.

The microbial metabolic quotient (qCO_2_) was calculated by:


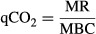
(1)

### Data analysis

Based on biologic measurements and field survey and monitoring, a total of 21 variables were derived and used in the analysis of site factors on soil microbial community structures, including the slope and cross-section area at breast height (CAB) for capturing potential influences of topography and tree stands; soil physiochemical properties consisting of *T*_soil10_, %SWC, pH, SOC, DOC, MBC, TN, NH_4_-N, NO_3_-N, C to N ratio in soil (C/N_soil_), and clay content (%Clay); biotic factors consisting of FR, litter mass, litter lignin content (%lignin_litter_), root lignin content (%lignin_root_), litter C to N ratio (C/N_litter_), root C to N ratio (C/N_root_), litter lignin to N ratio (lignin/N_litter_), and root lignin to N ratio (lignin/N_root_).

Redundancy analysis (RDA) was performed on data of relative abundance of individual PLFAs, expressed as % mole of the total, and site biotic and abiotic variables for determining the interrelationships between soil microbial composition and environmental conditions (Andersen et al. 2010; Thoms et al. 2010). We also used RDA to determine the linkage between soil microbial community types and the specific activities of soil extracellular enzymes involved in SOC acquisition and oxidation. The forward selection procedure in RDA, based on Monte Carlo permutation with 499 iterations, was performed to determine the most significant discriminating variables on the composition of individual PLFAs and the specific soil extracellular enzyme activities, and the significant variables (*P *< 0.05) were used in the final analyses. Significance tests for RDA were made using CANOCO software for Windows 4.5 (Biometris-Plant Research international, Wageningen, The Netherlands). All data were log-transformed before being used in the analyses (Thoms et al. 2010; Ushio et al. 2010; Wu et al. 2011).

Path analysis was conducted to examine the controls of biotic and abiotic factors on soil microbial composition and the regulatory pathway of changes in SOC relating to microbial community structure and function. This was made by constructing structural equation models, known as the path models, based on the conceptual model shown in Fig. 1. Structural equation modeling (SEM) is an advanced multivariate statistical technique that allows for hypotheses testing of complex path-relation networks (Grace et al. 2007). Path models were established and separately tested based on hypothetical connections between the dominant biotic and abiotic site factors and the structural attributes of soil microbial communities, as well as the linkage of the soil microbial community types with the function as represented by activities of soil extracellular enzymes involved in SOC transformation (i.e., C acquisition) and turnover (i.e., C oxidation). The soil temperature, soil water content (%), soil clay content (%), SOC content (%), and soil C to N ratio (C/N_soil_) were retained to represent microclimate and soil physicochemical properties, and the fine root biomass to represent plant factor. In the path model depicting the hypothesis on regulatory pathway of SOC dynamics, the MBC was considered as an early indicator of changes in SOC (Zhang et al. 2013); the abundance of bacteria and saprophytic fungi was retained to represent the structural attributes of soil microbial community, which correspond to an increase in C availability and recalcitrant compound decomposition, respectively (Strickland and Rousk 2010; Bárcenas-Moreno et al. 2011; Cusack et al. 2011), while the specific activities of the hydrolytic enzyme β-glucosidase and the oxidative enzyme phenol oxidase were retained to represent microbial function driving the C acquisition and the oxidation of complex C compounds, respectively (Cusack et al. 2011).

The normality of data distribution was examined for heteroscedasticity, and all bivariate relationships were checked for signs of nonlinearities before the SEM analysis. These analyses were performed by using the maximum-likelihood estimation procedures of Amos 17.0 (SPSS Inc., Chicago, IL). The adequacy of model fitting was determined by several tests, including the χ^2^ test (*P *> 0.05, CMIN/df < 2), the goodness of fit (GFI) (values >0.8 and <1), the root square mean error of approximation (RMSEA) (values <0.05), and the comparative fit index (CFI) (values >0.9) (Grace et al. 2010; Jassey et al. 2013; Zhang et al. 2013). To obtain the most parsimonious model, the nonsignificant indicators and pathways were eliminated in the final path model. For each set of analysis, *R*^2^ values were obtained for each dependent matrix, representing the proportion of total variance explained by the model (Grace et al. 2010).

## Results

### Controls of site factors on soil microbial composition

Among the 21 biotic and abiotic site variables, nine were found to relate significantly with the microbial composition, including % SWC, SOC, *T*_soil10_, % Clay, FR, C/N_soil_, C/N_litter_, NH_4_-N, and NO_3_-N (Table 1). The first two components of RDA axes explained 73.5% of the variance in the relationship between soil microbial composition and the nine selective site factors.

**Table 1:** Marginal and conditional effects of site factors on soil microbial composition obtained from the summary of forward selection in Redundancy analysis (RDA).

Variables	Lambda-A^1^	Lambda-B^2^	*P*^3^	*F*-ratio^4^
Soil water content (%SWC, w/w)	0.22	0.22	**0.002**	14.54
Soil organic carbon (SOC)	0.19	0.14	**0.002**	11.71
Soil temperature at 10 cm depth (*T*_soil10_)	0.19	0.07	**0.002**	6.21
Soil clay content (%Clay)	0.16	0.05	**0.002**	4.55
Fine root mass (FR)	0.16	0.04	**0.014**	3.49
Soil carbon to nitrogen ratio (C/N_soil_)	0.13	0.02	**0.020**	2.77
Nitrate nitrogen (NO_3_-N)	0.17	0.02	0.146	1.63
Litter carbon to nitrogen ratio (C/N_litter_)	0.12	0.02	0.136	1.62
Ammonium nitrogen (NH_4_-N)	0.18	0.00	0.962	0.32

Bold values indicate significant effects at *P* < 0.05.

1 Describe marginal effects, which show the variance explained when the variable is used as the only factor.

2 Describe conditional effects, which show the additional variance each variable explains when it is included in the model.

3 The level of significance corresponding to Lambda-B when performing Monte Carlo test at the 0.05 significance level.

4 The Monte Carlo test statistics corresponding to Lambda-B at the 0.05 significance level.

More than half of the variations in the microbial composition are explainable jointly by the nine factors (sum of Lambda-B in Table 1). Forward selection of the nine factors in the RDA ordinations showed that the microbial composition was primarily influenced by %SWC, SOC, *T*_soil10_, % clay content, FR, and C/N_soil_ (Table 1). Despite the relatively high marginal effect, NH_4_-N had negligible conditional effect on soil microbial composition.

**Figure 2:**
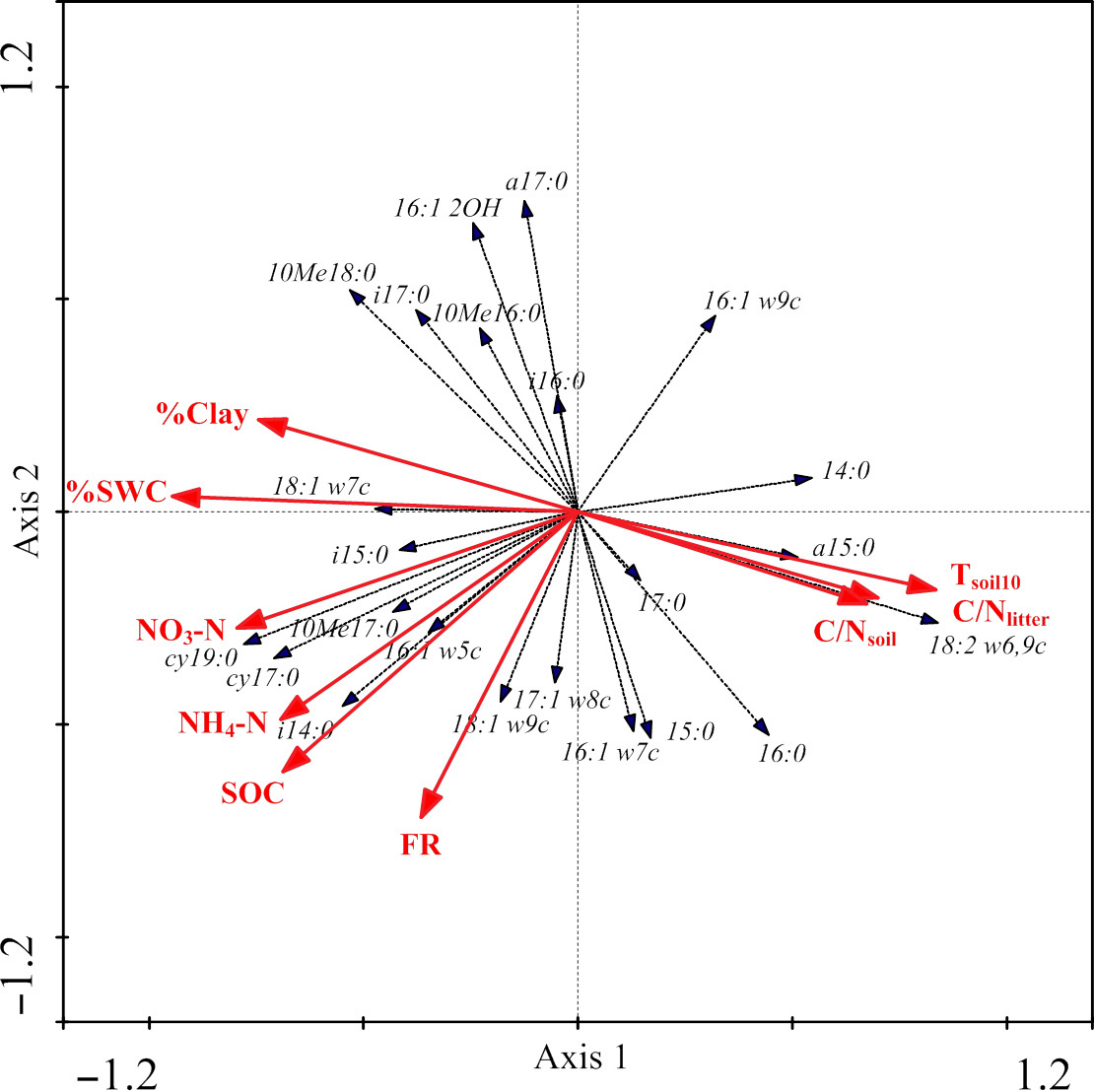
Redundancy analysis (RDA) ordination biplot of individual phospholipid fatty acids (PLFAs) and dominant site factors. *T*_soil10_, soil temperature at 10 cm depth; C/N_litter_, litter carbon to nitrogen ratio; C/N_soil_, soil carbon to nitrogen ratio; FR, fine root mass; SOC, soil organic carbon; NH_4_-N, ammonium nitrogen; NO_3_-N, nitrate nitrogen; %SWC, gravimetric soil water content (w/w); % Clay, soil clay content.

The RDA ordination biplot shows specific associations between the dominant site factors and individual PLFAs (Fig. 2). % SWC and % clay form positive associations predominantly with the Gram-negative bacteria 18:1ω7c and the Gram-positive bacteria i15:0, and negative associations with the saprophytic fungi 18:2ω6,9c, the Gram-positive bacteria a15:0, and the nonspecific PLFA 14:0; whereas *T*_soil10_, C/N_soil_, and C/N_litter_ are associated with the same PLFAs but in reversed pattern. FR is positively associated with the nonspecific PLFAs 18:1ω9c and 17:1ω8c and negatively associated with the nonspecific PLFA 16:1ω9c. SOC, NH_4_-N, and NO_3_-N are positively associated with the Gram-positive bacteria i14:0 and i15:0, the Gram-negative bacteria cy17:0 and cy19:0, the arbuscular mycorrhizal fungi 16:1ω5c, and the actinomycetes 10Me17:0, and negatively associated with the nonspecific PLFAs 16:1ω9c and 14:0. There are large numbers of PLFAs in various microbial communities not apparently associated with any of the dominant site factors (Fig. 2).

### Linkage between soil microbial community structure and function

**Figure 3:**
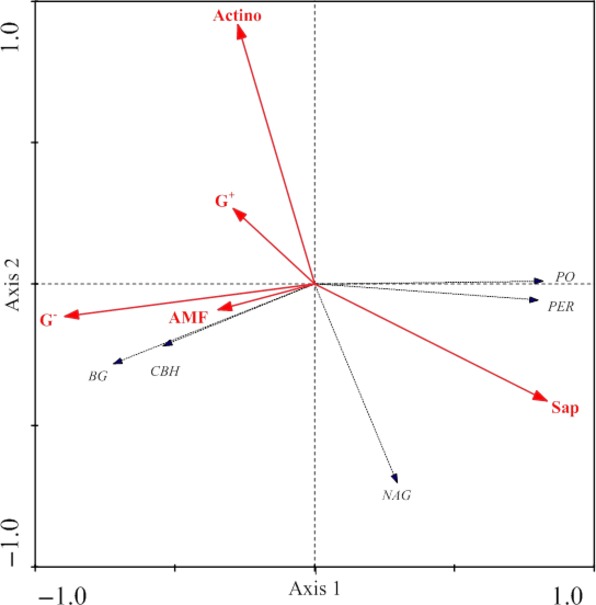
Redundancy analysis (RDA) ordination biplot of the abundance in five soil microbial community types and the specific activities of five soil extracellular enzymes. Sap, saprophytic fungi; G^+^, Gram-positive bacteria; G^−^, Gram-negative bacteria; AMF, arbuscular mycorrhiza fungi; Actino, actinomycetes; BG, β-glucosidase; CBH, cellobiohydrolase; NAG, N–acetyl-β-glucosaminidase; PO, phenol oxidase; PER, peroxidase.

In the RDA ordination biplot, axis 1 and axis 2 explained 84.7 and 11.9%, respectively, of the variance in the relationship between the specific activities of soil extracellular enzymes and the abundance in the five soil microbial community types (Fig. 3). The five soil microbial types jointly explained 66% of variations in the specific activities of the five soil enzymes assessed (sum of Lambda-B in Table 2), with Gram-negative bacteria, saprophytic fungi, and arbuscular mycorrhizal fungi appearing to be the most dominant controls (Table 2). The abundance in saprophytic fungi is positively associated primarily with the specific activities of phenol oxidase and peroxidase, and secondarily with NAGase, whereas Gram-negative bacteria and arbuscular mycorrhizal fungi positively correspond with the abundance of β-glucosidase and cellobiohydrolase. The abundance of actinomycetes is negatively associated with NAGase (Fig. 3).

**Table 2:** Marginal and conditional effects of soil microbial community types on specific activities of soil extracellular enzymes involved in SOC transformation and turnover obtained from the summary of forward selection in Redundancy analysis (RDA).

Variables	Lambda-A^1^	Lambda-B^2^	*P*^3^	*F*-ratio^4^
Gram-negative bacteria (G^−^)	0.44	0.44	**0.002**	40.72
Saprophytic fungi (Sap)	0.40	0.13	**0.002**	15.66
Actinomycetes (Actino)	0.11	0.08	**0.002**	10.87
Arbuscular mycorrhiza fungi (AMF)	0.07	0.00	0.546	0.71
Gram-positive bacteria (G^+^)	0.06	0.01	0.552	0.62

Bold values indicate significant effects at *P* < 0.05.

1 Describe marginal effects, which show the variance explained when the variable is used as the only factor.

2 Describe conditional effects, which show the additional variance each variable explains when it is included in the model.

3 The level of significance corresponding to Lambda-B when performing Monte Carlo test at the 0.05 significance level.

4 The Monte Carlo test statistics corresponding to Lambda-B at the 0.05 significance level.

### Regulatory pathway of soil microbial community structure

**Figure 4:**
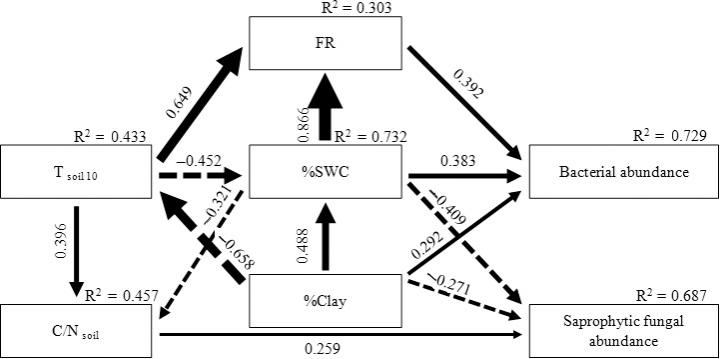
Path model depicting the regulatory pathway of the controls of abundance in bacterial and saprophytic fungal communities by dominant site factors. Numbers on arrows are standardized direct path coefficients. *R*^2^ value represents the proportion of total variance explained for the specific dependent variable. Dash-line arrows indicate negative effects. *T*_soil10_, soil temperature at 10 cm depth; C/N_soil_, soil carbon to nitrogen ratio; FR, fine root mass; %SWC, gravimetric soil water content (w/w); % Clay, soil clay content.

The path model on the controls of soil microbial community structure by dominant site factors well passed all the statistical tests on adequacy (*χ*^2^ = 2.8, *P *= 0.944, CMIN/df = 0.355; GFI = 0.985; CFI = 1.000; RMSEA < 0.001) and explained 72.9 and 68.7%, respectively, of the variance in the abundance of the bacterial and saprophytic fungal communities. Based on the path analysis, the abundance of soil bacterial community is directly and positively controlled by FR, %SWC, and %clay; whereas the abundance of saprophytic fungi community is directly and positively controlled by C/N_soil_ and negatively by %SWC and %clay (Fig. 4).

Apart from the direct controls, the path analysis also shows strong indirect effects of site factors by interacting with each other, resulting in cascade effects on the abundance of soil bacterial and saprophytic fungal communities (Table 3). Taking into consideration of both direct and indirect effects, % SWC exerts strongest total positive control on the abundance of soil bacterial community, followed by % clay and FR in descending order, whereas the abundance of soil saprophytic fungal community is most strongly and negatively affected by % clay content, followed by % SWC (Table 3). *T*_soil10_ has a strong indirect positive effect on the abundance of soil saprophytic community.

**Table 3:** Direct, indirect, and total path coefficients of the controls of dominant site factors on abundance of bacterial and saprophytic communities.

Factors	Effect	State variables							
		S (*T*_soil10_)	S (%SWC)	S (C/N_soil_)	S (FR)	S (Bacteria)	S (Sap)
F (% Clay)	Direct	−0.658	0.488	0	0	0.292	−0.271
	Indirect	0	0.297	−0.513	0.253	0.400	−0.454
	Total	−0.658	0.785	−0.513	0.253	0.692	−0.725
F (*T*_soil10_)	Direct	0	−0.452	0.396	0.649	0	0
	Indirect	0	0	0.146	−0.391	−0.072	0.325
	Total	0	−0.452	0.542	0.258	−0.072	0.325
F (%SWC)	Direct	0	0	−0.321	0.866	0.383	−0.409
	Indirect	0	0	0	0	0.340	−0.083
	Total	0	0	−0.321	0.866	0.723	−0.492
F (C/N_soil_)	Direct	0	0	0	0	0	0.259
	Indirect	0	0	0	0	0	0
	Total	0	0	0	0	0	0.259
F (FR)	Direct	0	0	0	0	0.392	0
	Indirect	0	0	0	0	0	0
	Total	0	0	0	0	0.392	0

% Clay, soil clay content; *T*_soil10_, soil temperature at 10 cm depth; %SWC, soil water content; C/N_soil_), soil carbon/nitrogen ratio; FR, fine root mass; Bacteria, abundance of bacterial community; Sap, abundance of saprophytic fungal community.

### Regulatory pathway of soil carbon transformation and turnover

**Figure 5:**
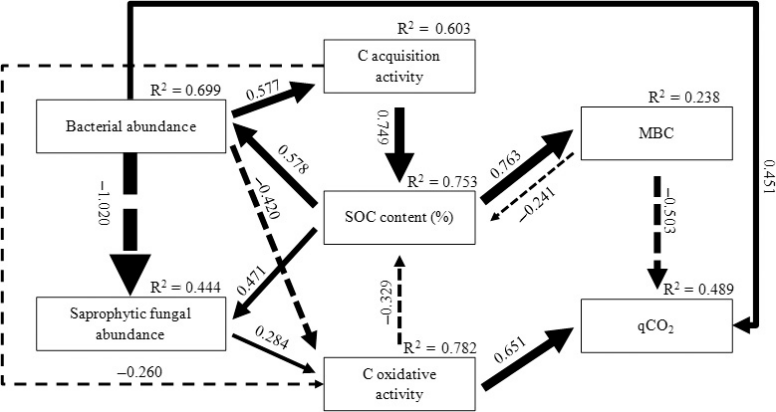
Path model depicting the regulatory pathway of the controls of soil organic carbon (SOC), microbial biomass carbon (MBC), and microbial metabolic quotient (qCO_2_) by the structural attributes of soil microbial community and specific activities of soil extracellular enzymes involved in C acquisition and oxidation. Numbers on arrows are standardized direct path coefficients. *R*^2^ value represents the proportion of total variance explained for the specific dependent variable. Dash-line arrows indicate negative effects. C acquisition is represented by the activity of β-glucosidase; C oxidation is represented by the activity of phenol oxidase.

The path model on the regulatory pathway of soil C-related variables also well passed all the statistical tests on adequacy (*χ*^2^ = 4.0, *P* = 0.776, CMIN/df = 0.577; GFI = 0.979; CFI = 1.000; RMSEA < 0.001) and explained 75.3, 23.8, and 48.9%, respectively, of the variance in SOC, MBC, and qCO_2_. The path analysis points to direct and strong positive control of C acquisition activity by abundance in soil bacterial community, whereas the C oxidative activity is directly controlled by a positive effect of abundance in soil saprophytic fungal community and by negative effects of abundance in soil bacterial community (Fig. 5). SOC imposes strong positive effects on the abundance of both bacterial and saprophytic fungal communities as well as MBC and is positively regulated by the C acquisition activity and negatively regulated by the C oxidative activity. Both the abundance of soil bacterial community and C oxidative activity imposes direct and strong positive effects on qCO_2_.

When considering also the indirect effects, the C acquisition activity is most strongly and positively controlled by the abundance in soil bacterial community, with SOC exerting a strong indirect positive effect; the C oxidative activity is mostly strongly and negatively controlled by the abundance in soil bacterial community, followed by C acquisition activity, with SOC exerting a strong indirect positive effect (Table 4). SOC is most strongly regulated by the total positive effect of C acquisition activity, followed by strong indirect effect of the abundance in soil bacterial community; C oxidative activity and MBC impose strong total negative effects on SOC. MBC is positively regulated by total effects of SOC and C acquisition activity and indirect effect of the abundance in soil bacterial community and negatively by C oxidative activity. qCO_2_ is strongly and positively regulated by the total effects of C oxidative activity and indirect effect of the abundance in soil saprophytic fungal community, and negatively by indirect effects of C acquisition activity and SOC and the total effects of the abundance in soil bacterial community and MBC.

**Table 4:** Direct, indirect, and total path coefficients of the controls of soil organic carbon (SOC), microbial biomass carbon (MBC), and microbial metabolic quotient (qCO_2_) by abundance of soil bacterial and saprophytic fungal communities via impacts on specific activities of extracellular enzymes involved in carbon acquisition and oxidation.

Factors	Effect	State variables								
		S (Bacteria)	S (Sap)	S (C_acquisition_)	S (C_oxidation_)	S (SOC)	S (MBC)	qCO_2_
F (Bacteria)	Direct	0	−1.020	0.577	−0.420	0	0	0.451
	Indirect	0.056	−0.104	0.291	−0.757	0.876	0.668	−0.875
	Total	0.506	−1.124	0.868	−1.177	0.876	0.668	−0.424
F (Sap)	Direct	0	0	0	0.284	0	0	0
	Indirect	−0.066	0.014	−0.038	0.042	−0.115	−0.087	0.226
	Total	−0.066	0.014	−0.038	0.326	−0.115	−0.087	0.226
F (C_acquisition_)	Direct	0	0	0	−0.360	0.749	0	0
	Indirect	0.591	−0.121	0.341	−0.271	0.274	0.780	−0.538
	Total	0.591	−0.121	0.341	−0.631	1.023	0.780	−0.538
F (C_oxidation_)	Direct	0	0	0	0	−0.329	0	0.651
	Indirect	−0.233	0.048	−0.134	0.146	−0.074	−0.308	0.145
	Total	−0.233	0.048	−0.134	0.146	−0.403	−0.308	0.796
F (MBC)	Direct	0	0	0	0	−0.241	0	−0.503
	Indirect	−0.171	0.035	−0.099	0.107	−0.055	−0.226	0.106
	Total	−0.171	0.035	−0.099	0.107	−0.296	−0.226	−0.397
F (SOC)	Direct	0.578	0.471	0	0	0	0.763	0
	Indirect	0.100	−0.616	0.408	−0.445	0.226	0.173	−0.441
	Total	0.708	−0.145	0.408	−0.445	0.226	0.936	−0.441

Bacteria, abundance of bacterial community; Sap, abundance of saprophytic fungal community; C_acquisition_, C acquisition activity (represented by the activity of β-glucosidase); C_oxidation_, C oxidative activity (represented by the activity of phenol oxidase); MBC, microbial biomass carbon; SOC, soil organic carbon; qCO_2_, microbial metabolic quotient.

## Discussion

A sound mechanistic understanding on the controls of biotic, climatic, and edaphic factors on soil carbon dynamics is a prerequisite for achieving a realistic prediction of changes in the regional soil carbon sink in response to changing environment. It is generally understood that SOC in forest ecosystems originates predominantly from decomposing plant litter and that the transformation and turnover of plant-derived organic carbon are mediated primarily by soil microbial communities (Singh et al. 2010; Schmidt et al. 2011). As microbial communities in soils act to either stabilize or turning over plant-derived organic carbon (Liang et al. 2011; Miltner et al. 2011), it has long remained a challenge to directly link the soil microbial attributes with the capacity of soil carbon sequestration. Despite a profound progress over the past two decades in elucidating the microbial community structure as affected by site or environmental conditions in forest soils (Högberg et al. 2007; Ushio et al. 2008; Thoms et al. 2010), associating the soil microbial structure with functioning in driving soil carbon transformation and turnover still occurs as an unresolved issue. In this study, by measuring a suite of complex site factors and soil microbial-related attributes under field conditions and applying multivariate analysis techniques, we examined ecosystem-level controls on soil microbial community structure and the linkage between microbial structure and function in soil carbon processes. Results show that at ecosystem level, factors relating to soil microclimate and fertility are the major controls on soil microbial structure and that the soil bacterial community and saprophytic community play distinct roles in mediating soil carbon processes in temperate forest ecosystem of the region.

Research to date has uncovered some of the regulations of soil microbial community composition by individual biotic and abiotic factors and/or their interactive effects (Bezemer et al. 2006; Thoms et al. 2010; Brockett et al. 2012). However, results are inconsistent concerning the critical drivers of differences in soil microbial structure. While the study of Högberg et al. (2007) shows that soil C to N ratio and pH are the major factors associated with variations in soil microbial community structure, the study of Mitchell et al. (2010) suggests that plant community composition better predicts changes in microbial community composition than soil properties. Thoms et al. (2010) found that plant community directly and indirectly affected soil microbial community composition through influences on the amount and quality of litter and soil physicochemical properties. Here, we show that soil water, SOC, soil temperature, soil clay content, fine root mass, and soil C to N ratio are all significant drivers of variations in soil microbial community composition. We found that soil water was positively associated with the abundance in bacterial community, especially the Gram-negative bacteria community, whereas soil temperature was positively associated with the abundance in saprophytic fungal community and negatively with the abundance in bacterial community. Both soil temperature and soil water have been identified as primary drivers of variations in soil microbial community structure in other studies (Hackl et al. 2005; Djukic et al. 2010; Brockett et al. 2012).

Factors relating to soil fertility, that is, SOC, NH_4_-N, NO_3_-N, and soil C to N ratio, were also found to correlate with soil microbial community structure. Bacteria are typically found in soils with rich SOC, whereas the relative abundance of saprophytic fungal community tends to increase with decreasing soil fertility (Grayston et al. 2004; Grayston and Prescott 2005; Franklin and Mills 2009; Katsalirou et al. 2010; Wu et al. 2011). The C to N ratio in both soil and litter was strongly and positively associated with saprophytic fungi in this study, consistent with the findings of positive correlation between the soil C to N ratio and the fungal to bacterial ratio by others (e.g., Högberg et al. 2007; Fierer et al. 2009). Our finding that high soil clay content favors the abundance of bacterial community and constrains the abundance of saprophytic fungal community is well supported by literature (e.g., Högberg et al. 2007; Lamarche et al. 2007; Fierer et al. 2009). Fine root mass, as a biotic factor, was found to associate mainly with nonspecific PLFAs. The influence of plant fine root mass on soil microbial community structure has been considered to involve several mechanisms, including alteration of soil microclimate, for example, changing soil water by uptake activity or modifying soil physical structure by root penetration, etc. (Prescott and Grayston 2013), and production of root litter (Loranger-Merciris et al. 2006; Ushio et al. 2008) and root extrudes (Kuzyakov and Donmanski 2000; Jones et al. 2004) as energy sources of soil microbial communities.

Several recent studies have demonstrated that soil pH is among the primary site factors determining soil microbial community structure (Högberg et al. 2007; Sinsabaugh et al. 2008). But in the current study, soil pH was not identified as a significant explanatory variable in the variations of soil microbial community composition. The narrow range of soil pH at our study sites (i.e., pH value 4.14–4.73) and inclusion of more dominant site factors may contribute to this discrepancy.

The nine dominant site factors explained only 58% of the total variations in soil microbial community composition. The relatively low predictive power overall suggests there are other unaccounted factors influencing the microbial community composition. The unexplained variances may possibly due to the interference of root exudates and growth regulators (Pires et al. 2012), condensed tannins (Ushio et al. 2010), total phenolic compounds (Ushio et al. 2010), and factors associated with soil type and structure (Girvan et al. 2003; Wakelin et al. 2008).

Changes in the soil microbial community composition are likely to result in shift in the function of microbial communities, thus altering soil biologic and physiochemical processes (Waldrop and Firestone 2006). Increases in the abundance of soil microbial communities that produce hydrolytic enzymes facilitate C acquisition in support of soil microbial primary metabolism (Cusack et al. 2011), whereas increases in the oxidative enzyme-producing microbial communities, mainly as saprophytic fungi, help with degrading complex compounds (Sylvia et al. 2004). An explicit understanding on the relationship between microbial community structure and function is crucial for predicting how shifts in microbial community structure may lead to changes in soil processes (Weand et al. 2010). In particular, identification of specific soil microbial attributes driving SOC transformation, and turnover is a significant step forward for gaining mechanistic understanding on the control of soil carbon sink and carbon sequestration potential in forest ecosystems (Lucas et al. 2007; Acosta-Martínez et al. 2010). With the help of RDA ordination, which is a well-known multivariate analysis technique for discriminating the relative effects when multiple factors are involved in regulating a specific process, we were able to establish the relative linkage between soil microbial community types and functional-specific soil extracellular enzymes. We found that the relative abundance in the Gram-negative bacteria and arbuscular mycorrhizal fungi communities was associated with the specific activities of hydrolytic enzymes involved in C acquisition (i.e., β-glucosidase and cellobiohydrolase), whereas the relative abundance in the saprophytic fungi community was correlated with the specific activities of soil enzymes involved in degradation of chitin (i.e., NAGase) and lignin (i.e., phenol oxidase and peroxidase). Gram-negative bacteria generally grow fast to compete for simple organic compounds (Waldrop et al. 2000) and were found in this study to significantly relate to the specific activity of β-glucosidase involved in the breakdown of complex organic compounds into small molecules substrates in favor of C acquisition through microbial community growth. Gram-positive bacteria were positively correlated with cellobiohydrolase responsible for degrading complex compounds (Waldrop et al. 2000; Bell et al. 2009). The saprophytic fungi possess the physiological capacity to produce phenol oxidase and peroxidase essential in lignin depolymerization (Colpaert and Laere 1996; Courty et al. 2008), as well as to produce NAGase involved in chitin degradation (Miller et al. 1998; Burke et al. 2011).

Our results suggest that the complex interactions between the biotic and abiotic site factors may further propagate in affecting the soil microbial community function involved in SOC transformation and/or turnover through modification of the bacterial and saprophytic fungal community structure, shifts in the allocation of plant-derived organic carbon to microbial biomass, and regulation of microbial activities. Moreover, factors affecting soil microbial community structure may also impose direct and/or indirect impacts on the microbial function, enhancing or weakening the overall controls of soil microbial community function by individual site factors.

We used the specific activity of the soil extracellular enzymes (i.e., enzymatic per unit SOC) to assess the dynamics of SOC transformation and/or turnover in path analysis. Soil bacteria community was identified to strongly regulate SOC storage although increasing the microbial C acquisition activity. Saprophytic fungi community contributes to SOC turnover by increasing the microbial C oxidative activity. Extracellular enzyme activity in soils has been studied for more than a century with a goal of understanding the biochemistry of decomposition and nutrient cycling. Extracellular enzymes are widely produced by different groups of soil microorganisms, and some of them can be used as indicators of the presence or activity of specific microbial taxa (Baldrian 2009). They provide useful insights into the mechanisms of microbial sensitivity to environment change (Cusack et al. 2011). The most widely assayed enzymes are those involved in the degradation of cellulose and lignin, the most abundant components of plant litter (Cusack et al. 2011). Other commonly measured enzymes hydrolyze proteins, chitin, and peptidoglycan, which are the principal reservoirs of organic N (Caldwell 2005). However, none of the soil enzyme assay techniques is able to completely reflect the in situ conditions and to measure the actual rates of enzymatic reactions (Toberman et al. 2008). Quantitative PCR of gene sequences or mRNA transcripts was recently used for quantification of some enzyme-encoding genes, including phenoloxidase–laccase (Hassett et al. 2009; Lauber et al. 2009). However, the connection between transcript or gene copy level and enzyme activity is indirect and is biased by genes encoding inactive molecules (Baldrian 2009).

In summary, our study reveals complex controls of soil carbon dynamics by various above-and below-ground processes and their interactive effects. Soil microbial community composition is strongly influenced by water, temperature, SOC, fine root mass, clay content, and C/N ratio in soils. Soil bacterial communities are strongly linked with the extracellular enzymes involved in carbon transformation, whereas saprophytic fungi are associated with activities of extracellular enzymes driving carbon oxidation. There exist complex interactions and linkage among plant traits, microenvironment, and soil physiochemical properties in affecting SOC via microbial regulations in temperate forests.
